# RANK/RANKL/OPG Axis Expression and Response to Anti-RANKL Therapy in Gnathic Giant Cell Lesions: A Systematic Review and Meta-Analysis

**DOI:** 10.3390/diseases14100360

**Published:** 2026-10-01

**Authors:** Darya Khalid Mahmood, Arivan Mahmood Hama, Ahmad Shoeb Hashmi

**Affiliations:** 1 Oral Pathology, Oral Diagnosis Department, College of Dentistry, University of Sulaimani, Sulaimani 46001, Iraq; 2 Department of Prosthodontics, College of Dentistry, University of Sulaimani, Sulaimani 46001, Iraq; arivan.hama@univsul.edu.iq; 3 Johns Hopkins Bloomberg School of Public Health, Johns Hopkins University, Baltimore, MD 21218, USA; gi6367@myamu.ac.in; 4 Department of Dental Research Cell, Dr. D. Y. Patil Dental College & Hospital, Dr. D. Y. Patil Vidyapeeth, Pune 202001, India

**Keywords:** central giant cell granuloma, peripheral giant cell granuloma, cherubism, aneurysmal bone cyst, immunohistochemistry, osteoprotegerin, RANKL, RANK, denosumab

## Abstract

Background/Objectives: Understanding the biological role of the receptor activator of nuclear factor-κB ligand (RANK), its ligand (RANKL), and the decoy receptor osteoprotegerin (OPG) is vital for clarifying the pathogenesis of gnathic giant cell-containing lesions and directing targeted clinical treatments. Methods: Following PRISMA 2020 guidelines and a registered PROSPERO protocol [CRD420261461026], we searched PubMed, Scopus, and Web of Science databases for human tissue-level or clinical denosumab studies (2003–2026). Quality was appraised using adapted NOS and JBI checklists. A fixed-effect inverse-variance meta-analysis pooled continuous RANK parameters between aggressive and non-aggressive CGCG subtypes. Results: Eleven primary tissue-marker studies (259 specimens; two previously considered studies were excluded as unpublished, non peer-reviewed sources) and 10 non-overlapping clinical cohorts/case reports (58 patients total: 55 from 7 multi-patient cohorts + 3 single-case reports [Hameed, O’Connell, Kawamura]). Mononuclear stromal cells represent the pathogenetic driver, secreting RANKL to recruit RANK-expressing mature giant cells. Meta-analysis (k = 2 studies) showed that raw membrane RANK-positive cell percentages did not differ significantly between aggressive and non-aggressive phenotypes (pooled Hedges’ g = 0.25, 95% CI −0.29–0.80, *p* = 0.36), while non-aggressive lesions showed a borderline, non-significant trend toward higher staining intensity-distribution (SID) scores (g = 0.55, 95% CI −0.00–1.11, *p* = 0.0506). Because this 95% CI crosses zero, and only two studies (k = 2) were pooled, this represents a non-significant trend rather than a statistically confirmed difference between aggressive and non-aggressive CGCG; both pooled estimates are, therefore, hypothesis-generating only. In clinical cohorts, denosumab achieved objective clinical and radiological tumor responses in all reported cases, enabling downstaging from disfiguring resection to conservative curettage. However, post-cessation recurrence was frequent, ranging from 37% in adults to 100% in pediatric cohorts, with pediatric patients at risk of rebound hypercalcemia. Conclusions: The RANK/RANKL/OPG axis, driven by RANKL-secreting mononuclear stromal cells, underlies osteolytic activity in gnathic giant cell lesions. Denosumab is an effective neoadjuvant bridge to jaw-preserving surgery but is not curative; a RANKL-independent, OPG-resistant SOFAT pathway has been hypothesized as one possible contributor to treatment failure, although this proposal is derived from a single external study and requires independent validation before any clinical inference is drawn. Close post-treatment metabolic and radiographic surveillance is warranted in pediatric patients. Prospective studies are needed to define optimal weaning protocols.

## 1. Introduction

The gnathic skeleton of the jaw is an active metabolic organ subjected to constant physiological mechanical stress, requiring highly synchronized and tightly coupled remodeling processes to maintain microstructural integrity and calcium homeostasis [1]. This continuous remodeling represents a coordinated cellular interaction between the bone-resorbing activity of hematopoietically derived multinucleated osteoclasts and the bone-forming capacity of mesenchymal lineage osteoblasts [2]. Under normal physiologic conditions, bone remodeling is regulated by RANK, its ligand RANKL, and receptor OPG, collectively termed the RANK/RANKL/OPG signaling pathway [3,4]. The binding of osteoblast-derived or stromal cell-derived RANKL to its receptor RANK on osteoclast precursors promotes their fusion, survival, and differentiation into mature, bone-resorbing multinucleated osteoclasts [5,6]. OPG, a decoy receptor secreted by osteoblasts, binds competitively to RANKL, preventing its interaction with RANK and, thereby, inhibiting bone resorption [4,7]. Imbalances in the local RANKL/OPG ratio are central to the pathogenesis of various osteolytic skeletal diseases, including giant cell-rich lesions [4,8].

Gnathic giant cell-containing lesions include a heterogeneous spectrum of non-neoplastic, reactive, and genetic osteolytic disorders, primarily CGCG, PGCG, cherubism, and ABC [9]. Despite their divergent clinical courses, these lesions exhibit marked histopathological overlap, characterized by abundant multinucleated giant cells (MGCs) embedded in a fibrous stroma consisting of spindle-shaped mononuclear stromal cells (MSCs), endothelial channels, and hemorrhagic foci [10]. While PGCG presents as an extraosseous, exophytic gingival nodule with minimal bone destruction, CGCG arises intraosseously and can demonstrate highly aggressive expansion, causing cortical perforation, tooth displacement, and severe root resorption [11]. Cherubism is a rare genetic disorder caused by gain-of-function mutations in the SH3BP2 gene, driving symmetrical bilateral multilocular expansion of the jaws in young children [12]. ABC represents a pseudocystic lesion characterized by blood-filled sinusoidal spaces lined by MGC-rich fibroblastic tissue [5]. Although CGCG, PGCG, cherubism, and ABC converge on this shared multinucleated giant cell and mononuclear stromal cell histomorphology, current evidence indicates that they arise through distinct pathogenetic mechanisms and, consequently, differ in biological behavior and expected treatment responses. CGCG and PGCG are generally regarded as reactive or hyperplastic processes without a defined clonal genetic driver; cherubism is a monogenic disorder caused by germline gain-of-function SH3BP2 mutations [12]; a substantial proportion of ABCs harbor clonal USP6 gene rearrangements that place USP6 under the control of a heterologous promoter, supporting a neoplastic rather than purely reactive origin for at least a subset of these lesions [13]. These differing origins likely contribute to differences in natural growth potential, recurrence behavior, and, potentially, responsiveness to RANKL-targeted therapy—and argue against treating CGCG, PGCG, cherubism, and ABC as a single uniform clinical entity despite their shared giant cell morphology. Over the last two decades, advances in translational bone pathology have revealed that local hyperactivation of the RANK/RANKL/OPG pathway represents the common molecular denominator driving the pathological osteolysis across these gnathic conditions [14].

Denosumab, a fully humanized monoclonal IgG2 antibody directed against human RANKL, has revolutionized the treatment of aggressive osteoclastic bone disorders, particularly giant cell tumor of bone (GCTB) [15]. Increasingly, denosumab has been applied off-label as a medical intervention for aggressive or recurrent gnathic giant cell lesions, in order to avoid disfiguring mandibular or maxillary resections [8,16].

This manuscript presents the first comprehensive systematic review and quantitative meta-analysis conducted to map the tissue expression of the RANK/RANKL/OPG pathway, specifically within giant cell-containing lesions of the jaws (CGCG, PGCG, cherubism, and ABCs) and correlate these pathogenetic markers with clinical responses to targeted anti-RANKL therapy (denosumab). The clinical significance of this systematic review and staining meta-analysis lies in its ability to bridge basic laboratory pathology with organ-preserving bedside medicine. By systematically evaluating tissue-level expression patterns and cellular localization of RANK, RANKL, and OPG, this paper defines the pathogenetic engines driving osteoclastogenesis. Furthermore, we analyze data from clinical cohorts to establish denosumab’s primary role as a neoadjuvant bridge to the downstaging of mutilating surgeries to conservative, low-morbidity enucleations. Crucially, we address post-treatment safety profiles—highlighting the high rate of metabolic recurrence and pediatric rebound hypercalcemia—and synthesize institutional weaning and surveillance protocols to optimize both jaw preservation and systemic patient safety.

## 2. Materials and Methods

### 2.1. Search Strategy and Study Selection

This systematic review adhered closely to the PRISMA 2020 (Preferred Reporting Items for Systematic Reviews and Meta-Analyses) framework [17], with the review protocol formally registered a priori on PROSPERO, the international prospective register of systematic reviews (ID CRD420261461026), where it is publicly accessible at https://www.crd.york.ac.uk/prospero/ (accessed on 22 September 2026). No amendments were made to the registered protocol or eligibility criteria after registration. The completed PRISMA 2020 checklist is provided in Supplementary File S1.

Electronic database searches were executed across PubMed/MEDLINE, Scopus, and Web of Science from database inception through August 2025. The search strategy utilized combinations of Boolean operators and MeSH terms, combining target markers (RANK, RANKL, OPG, receptor activator of nuclear factor-κB ligand, osteoprotegerin) with specific gnathic lesions (central giant cell granuloma, peripheral giant cell granuloma, giant cell lesion of the jaw, cherubism, aneurysmal bone cyst). The full search strategy for each database is provided in the Supplementary Materials File S2. Broad screening was conducted to ensure that eligible non-English-language publications would be evaluated via professional translation; no non English-language studies ultimately met the eligibility criteria. The search was updated until July 2026 and was conducted in duplicate by two authors (D.K.M., A.M.H.). Data extraction from eligible reports was, likewise, performed independently and in duplicate by two authors (D.K.M., A.M.H.) using a standardized, pre-piloted extraction form; discrepancies were resolved by discussion and, when unresolved, by consultation with the third author (A.S.H.).

### 2.2. PECO/PICO Framework

To establish a clear and transparent audit trail for study inclusion, the review questions and search parameters were defined using a structured Population, Exposure, Comparator, and Outcome (PECO/PICO) framework. as outlined below:

PECO/PICO Elements’ Methodological Definition/Criteria
Population: Histopathologically confirmed giant cell-containing lesions of the jaws: central giant cell granuloma (CGCG), peripheral giant cell granuloma (PGCG), and—where reported alongside these—cherubism and aneurysmal bone cyst (ABC) as related giant cell-containing entities. Jaw/gnathic lesions only.
Intervention/Exposure: RANK (receptor activator of NF-κB), RANKL (RANK ligand), and OPG (osteoprotegerin) expression, assessed by immunohistochemistry (IHC) or in situ hybridization (ISH);
Comparator: Between-lesion comparison, where reported (e.g., CGCG vs. PGCG); within-lesion characterization where no direct comparator exists; and, for the therapy sub-question, pre- vs. post-treatment expression or expression stratified by treatment response;
Outcomes: (a) Proportion of RANK/RANKL/OPG-positive cases, staining extent/intensity score, or mean expression value ± SD; and (b) where reported in the same cohort, therapy administered and clinical/marker response to that therapy.

### 2.3. Eligibility Criteria

To ensure methodological consistency and prevent clinical confounding, strict eligibility boundaries were pre-specified. Studies were screened and selected according to the following criteria:

Inclusion Criteria:

(1) Original human tissue investigations (archival paraffin-embedded or fresh biopsy);
(2) Histopathologically confirmed central giant cell granuloma (CGCG), peripheral giant cell granuloma (PGCG), cherubism, or aneurysmal bone cyst (ABC) of the jaws/gnathic skeleton;
(3) Tissue expression of RANK, RANKL, and/or OPG assessed by immunohistochemistry (IHC) or in situ hybridization (ISH);
(4) Original clinical trials, prospective/retrospective cohorts, or institutional case series (comprising N ≥ 5 cases) reporting quantifiable or semi-quantifiable staining datasets;
(5) For the clinical therapy sub-question only: studies additionally reporting a treatment/intervention and corresponding clinical outcome or marker change linked to that treatment within the exact same cohort that also reports RANK/RANKL/OPG expression.

Exclusion Criteria:

(1) Giant cell tumor of long bones (GCTB) as the sole primary population, due to distinct anatomic and biological differences;
(2) Odontogenic cysts or tumors (e.g., ameloblastoma, keratocyst, dentigerous cyst) to prevent clinical overlap;
(3) Studies with fewer than five human cases (excluding single-patient case reports synthesized separately for the clinical therapy sub-question);
(4) Pure laboratory studies, animal models, or in vitro cellular lines without original primary human tissue specimen analysis;
(5) Consensus guidelines, and conference abstracts;
(6) Clinical therapy studies in CGCG/PGCG that do not assess RANK/RANKL/OPG expression (out of scope).

Titles and abstracts were screened by two reviewers (A.M.H. and A.S.H.) independently. Decisions for screening were made such that the reviewers were unaware of the other reviewer’s judgments until both had completed screening. Reports considered potentially eligible by either reviewer were retrieved in full text and independently assessed against the predefined eligibility criteria by the same reviewers; disagreements at both the title/abstract and full-text stages were resolved by discussion and, where necessary, adjudication by a third author (D.K.M.). Formal inter-rater agreement statistics (e.g., Cohen’s kappa) for the screening process were not calculated.

### 2.4. Methodological Stratification and Quality Appraisal

To prevent confounding from methodologically heterogeneous detection techniques, included publications were stratified into independent subgroups: Subgroup A (IHC evaluating protein expression), Subgroup B (ISH evaluating spatial mRNA localization), and Subgroup C (other, e.g., qPCR on tissue homogenates) [18]. Methodological quality and risk of bias (RoB) were independently evaluated by two reviewers (D.K.M and A.M.H). Observational comparative studies were appraised using the adapted Newcastle–Ottawa Scale (NOS) across selection, comparability, and exposure/outcome domains (maximum of 9 stars) [19]. Non-comparative case series and clinical case reports were appraised using the standardized Joanna Briggs Institute (JBI) Critical Appraisal Checklists [20]. The NOS was “adapted” in that only the Selection, Comparability, and Exposure/Outcome domains applicable to retrospective tissue-marker comparisons were scored (maximum 9 stars, rather than the 9–10-star maxima used for cohort or case-control designs in other fields); no JBI checklist items were modified from their published form. Disagreements in risk-of-bias scoring between the two reviewers were resolved by discussion and, where necessary, adjudication by the third author (A.S.H.). Formal inter-rater agreement statistics (e.g., Cohen’s kappa) for risk-of-bias scoring were not calculated.

### 2.5. Quantitative Staining Meta-Analysis

To evaluate the quantitative association between continuous tissue levels of cell-surface RANK expression and clinical aggressiveness, we pre-specified that only studies reporting continuous RANK outcomes using directly comparable staining and scoring protocols would be pooled quantitatively; all other tissue studies were synthesized narratively. Applying this rule, two studies met the criterion for quantitative pooling: Sergio et al. [3] (N = 37) and Taher et al. [21] (N = 21). Both studies assessed continuous outcomes—specifically, the raw percentage of RANK-positive cell expression and the semi-quantitative SID score (calculated as proportion of stained cells multiplied by staining intensity)—across clinically aggressive and non-aggressive CGCG cohorts [3,21]. Standardized mean differences (SMDs) with Hedges’ g correction for small sample sizes were pooled using inverse-variance weights, with significance determined at the alpha = 0.05 level [22,23]. Because only two studies met the strict criterion of employing identical staining and scoring protocols, heterogeneity (I^2^, Cochran’s Q) could not be formally estimated; the fixed-effect model was, therefore, adopted as the more conservative choice for this minimal evidence base, rather than because homogeneity had been demonstrated; the pooled estimates should be interpreted as hypothesis-generating rather than definitive. Standardized mean differences (Hedges’ g with 95% confidence intervals) were pooled using a fixed-effect inverse-variance model in RevMan v5.4. Both pooled studies reported means and standard deviations directly in their published tables; no data imputation, estimation from medians/IQRs, or unit conversion was required. Because only two studies met the pooling criterion, exploration of heterogeneity sources (e.g., subgroup analysis) and sensitivity analyses were not applicable and were not conducted; this is noted as a limitation in Section 4.

Given the small number of eligible studies for both the tissue-marker synthesis and the quantitative meta-analysis, risk of bias due to missing results (e.g., publication bias, selective outcome reporting) was not formally assessed with funnel plots or statistical tests, as such methods are underpowered and unreliable with fewer than ten studies per synthesis; this is stated here prospectively as a methodological limitation rather than post hoc. Specifically, publication-bias assessment (funnel plots and formal asymmetry tests such as the Egger or Begg test) was not performed, because fewer than 10 eligible studies contributed to each synthesis. Similarly, no formal certainty-of-evidence assessment (e.g., GRADE) was performed, given the predominantly descriptive and mechanistic nature of the tissue-marker synthesis and the small, non-randomized clinical evidence base; findings should, therefore, be interpreted as hypothesis-generating rather than as a graded body of evidence.

## 3. Results

### 3.1. Study Selection Flow and Literature Characteristics

**Figure 1:**
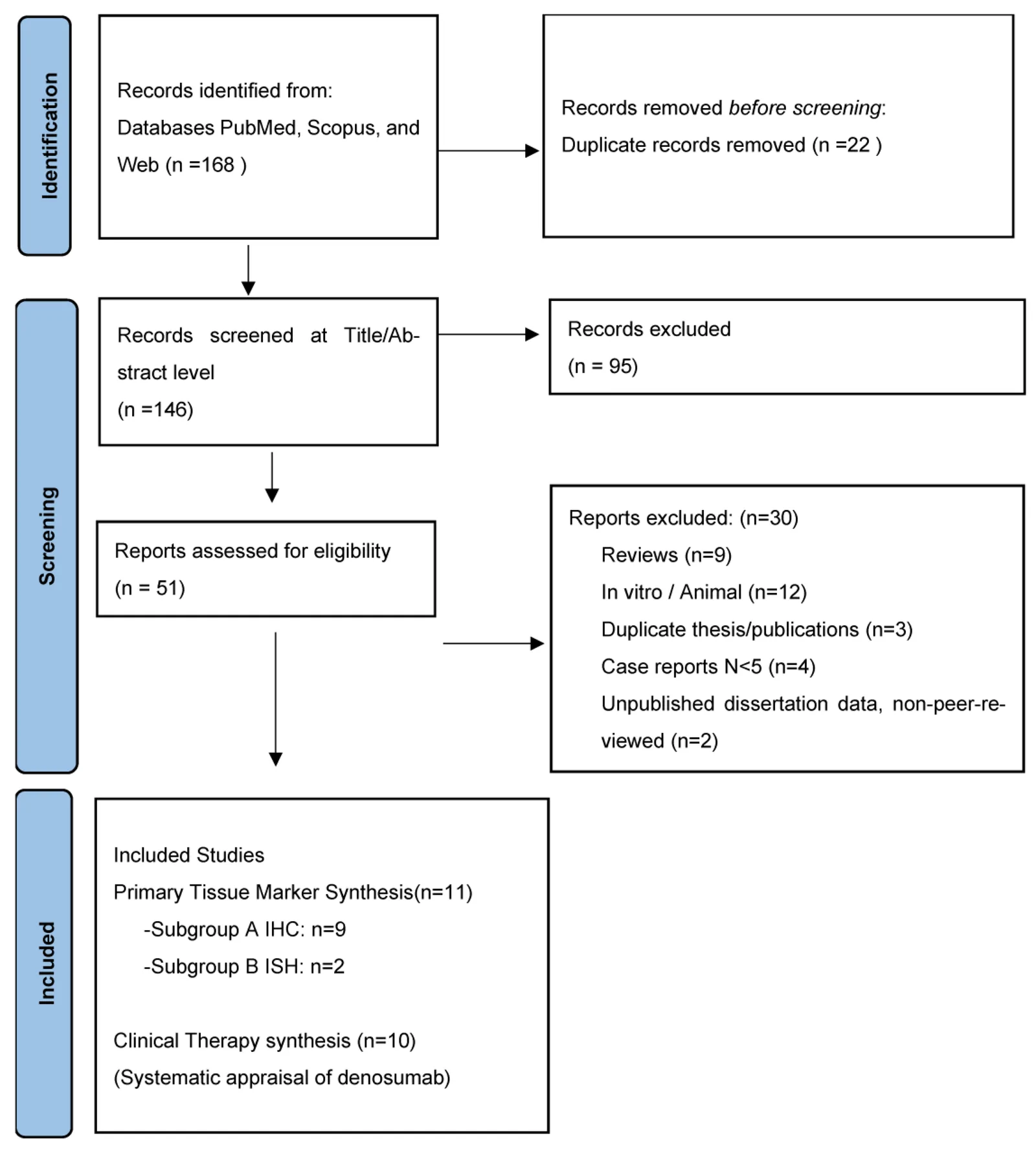
PRISMA 2020 Flow diagram of literature screening and selection. Flow chart documenting database screening from 168 initial records down to 11 primary human tissue studies (Subgroup A [IHC]: 9, Subgroup B [ISH]: 2) and 10 clinical denosumab therapy investigations (seven multi-patient cohorts and three single-case reports).

The systematic literature search retrieved a total of 168 records. After automatic duplicate removal, 22 duplicates were excluded, leaving 146 records for title/abstract screening. Abstract screening excluded 95 irrelevant or non-original articles. Fifty-one (51) candidate full-text reports were sought and retrieved for clinical and methodological eligibility. Detailed full-text review led to the exclusion of 30 papers (reviews: nine; animal or in vitro cellular models: 12; duplicate thesis/publications: three; case reports with N < 5 cases: four; unpublished dissertation-level data not meeting the pre-specified exclusion of consensus/non-peer-reviewed sources, Exclusion Criterion 5: 2—unpublished dissertation-level reports excluded under Criterion 5; not included in the numbered reference list). Ultimately, 11 primary human tissue-marker studies (nine IHC, two ISH) met the criteria for primary pathogenetic synthesis, and 10 clinical investigations evaluating denosumab were included for clinical outcomes synthesis (Figure 1, PRISMA flow diagram). Among the included papers, significant duplication of patient databases was identified within the clinical therapy studies from Westmead Hospital [16,24,25] and Amsterdam UMC [8]. To prevent patient double-counting, these duplicate databases were consolidated, leaving seven primary multi-patient clinical cohorts eligible for clinical outcome tabulation. The detailed study selection flow is visually detailed in Figure 1.

Key pathogenetic and methodological findings of the 11 included tissue-marker studies are summarized in Table 1.

### 3.2. Quality and Risk-of-Bias Assessment of Included Studies

Critical quality appraisal was conducted for all 21 included studies (11 primary tissue studies and 10 clinical therapy studies). For comparative cross-sectional or cohort designs (seven tissue studies), the adapted Newcastle–Ottawa Scale (NOS) was utilized (total stars = 9), with studies scoring ≥6 stars categorized as possessing a Low Risk of Bias (High Quality). For the remaining descriptive tissue case series (4 studies) and all 10 clinical investigations, the Joanna Briggs Institute (JBI) Critical Appraisal Checklists were applied (reported as percentages of criteria met). As detailed in Table 2, 16 of the 20 formally appraised studies possessed a Low Risk of Bias, while 4 presented a Moderate Risk of Bias. The primary methodological limitation across the comparative studies was the absence of cohort matching (for age and gender) in the comparability domain, with the sole exception of Morais et al. [26]. Within the tissue case series, the primary risk of bias was clinical imprecision due to extremely small sample sizes (e.g., N = 7 in Ahmed and Dunlap [6]). Additionally, a major technical gap, identified in around 85% of primary tissue studies, was the failure to report or control for tissue decalcification methods (such as EDTA versus aggressive hydrochloric acid), which compromises protein and mRNA integrity, risking false-negative staining. Among the 10 clinical studies, retrospective designs were universally prone to selection bias and 70% of investigations failed to report longitudinal pre- and post-treatment systemic bone turnover markers (PTH, CTX, P1NP), presenting a prominent risk of undocumented subclinical metabolic bone changes.

### 3.3. Compartment-Specific Cellular Localization of Axis Markers

Synthesizing the histopathological and molecular hybridization data from all 11 primary marker studies establishes a clear compartment-specific division of labor within the lesional microenvironment (Table 3). Multinucleated giant cells (MGCs) across CGCG, PGCG, cherubism, and ABC represent the mature resorptive effector population, expressing the receptor RANK on their cell membranes, along with osteoclastic functional enzymes (TRAP, V-ATPase, Cathepsin K, and MMP-9) [5,26]. Conversely, the proliferating, spindle-shaped mononuclear stromal cells (MSCs) and mononuclear round cells represent the highly proliferative lesional component (expressing PCNA and Ki-67) and serve as the primary diffuse source of pathogenetic RANKL secretion [6,7]. Recruited mononuclear pre-osteoclasts (CD68-positive) express RANK on their membranes and undergo fusion into MGCs under the local influence of stromal cell-derived RANKL. OPG decoy receptors are widely distributed across both mononuclear and multinucleated components, suggesting a local feedback loop; however, a statistically significant elevation of the local RANKL/OPG ratio in CGCG mononuclear stromal cells compared to PGCG correlates with cortical perforation and clinical aggressiveness.

### 3.4. Quantitative Meta-Analysis of Continuous RANK Tissue Expression

**Figure 2:**
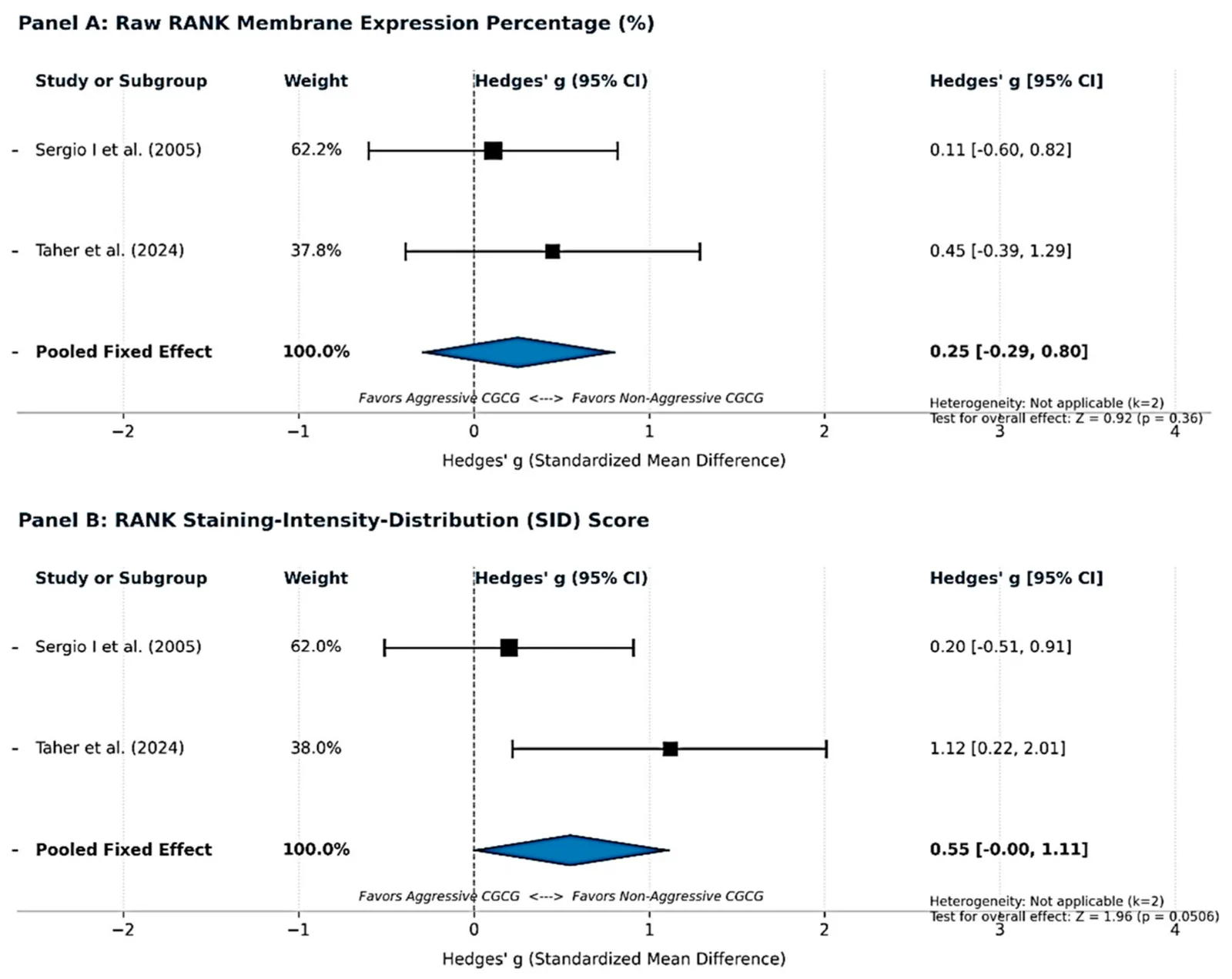
Fixed-effect meta-analysis of continuous tissue RANK staining outcomes. Fixed-effect inverse-variance pooling of standardized mean differences with Hedges’ g correction for continuous RANK outcomes between aggressive and non-aggressive CGCG. Panel (**A**): raw percentage of membrane RANK expression (%) is statistically indistinguishable between subtypes (pooled Hedges’ g = 0.25, 95% CI: −0.29 to 0.80, *p* = 0.36) [3,21]; and Panel (**B**): staining intensity-distribution (SID) score shows a non-significant trend favoring non-aggressive CGCGs (pooled Hedges’ g = 0.55, 95% CI: −0.00 to 1.11 *p* = 0.0506). This borderline *p*-value does not meet the conventional 0.05 threshold; thus, the comparison is not statistically significant. As the 95% CI spans zero, and only two studies were pooled (heterogeneity not estimable), this should be read as hypothesis-generating rather than a confirmed difference. In each panel, squares represent the standardized mean difference (Hedges’ g) for each individual study, with the horizontal line through each square denoting its 95% confidence interval; the diamond (rhombus) represents the pooled fixed-effect summary estimate, with the width of the diamond indicating the 95% confidence interval of the pooled effect.

To evaluate the quantitative association between continuous tissue levels of cell-surface RANK expression and the clinical behavior of CGCG of the jaws, we performed a targeted fixed-effect meta-analysis of the two homogeneous studies, employing identical staining and scoring protocols: Sergio et al. [3] (N = 37) and Taher et al. [21] (N = 21), totaling N = 58 cases. For Outcome A (raw percentage of RANK-positive cells), the individual standardized effect sizes were Hedges’ g = 0.11 (95% CI: −0.60 to 0.82) for Sergio et al. [3] and Hedges’ g = 0.45 (95% CI: −0.39 to 1.29) for Taher et al. [21]. The pooled fixed-effect standardized mean difference was Hedges’ g = 0.25 (95% CI: −0.29 to 0.80; *p* = 0.3599). This pooled estimate demonstrates that there is no statistically significant difference in raw cell-surface RANK expression between clinically aggressive and non-aggressive CGCG. For Outcome B (RANK SID score, which combines percentage staining with subjective color density), the individual effect sizes were Hedges’ g = 0.20 (95% CI: −0.51 to 0.91) for Sergio et al. [3] and Hedges’ g = 1.12 (95% CI: 0.22 to 2.01) for Taher et al. [21]. The pooled fixed-effect standardized mean difference was Hedges’ g = 0.55 (95% CI: −0.00 to 1.11; Z-statistic = 1.955; *p*-value = 0.0506). Although the *p*-value approaches the conventional 0.05 threshold, the 95% confidence interval spans zero; this should, therefore, be interpreted as a non-significant trend toward higher RANK receptor density and staining intensity in non-aggressive lesions, not as a statistically confirmed difference, particularly given that only two studies contributed to this pooled estimate. Accordingly, no statistically confirmed difference in RANK SID score between aggressive and non-aggressive CGCG can be claimed from this pooled analysis; the result is a non-significant trend and is hypothesis-generating only. This outcome is highly driven by the significant clinical differences reported by Taher et al. [21], where the non-aggressive cohort displayed a significantly higher SID score (7.50 ± 2.39 vs. 5.12 ± 1.76, *p* = 0.02088). In contrast, aggressive CGCG in Taher’s cohort was characterized by a massive osteoblastic bone-rebuilding response, with osteocalcin expression being significantly elevated in aggressive cases (30.16% vs. 8.58%, *p* = 0.0232), indicating that clinically aggressive lesions maintain dynamic osteogenic potential. These findings are visually compiled in Figure 2. As only two studies contributed to each pooled outcome, investigation of heterogeneity sources and sensitivity analyses were not applicable and were not performed; risk of bias due to missing results and a formal certainty-of-evidence grading were, likewise, not assessed for this synthesis, for the reasons stated in Section 2.5.

### 3.5. Clinical Efficacy, Recurrence, and Safety of Targeted RANKL Inhibition

To analyze the clinical predictive value of the RANK/RANKL/OPG pathogenetic pathway, we qualitatively synthesized the therapeutic outcomes of denosumab, a humanized monoclonal antibody designed to block RANKL, across seven distinct, multi-patient cohorts (totaling 55 patients) after excluding overlapping institutional databases to prevent patient double-counting. Subcutaneous denosumab achieves a uniform 100% objective clinical and radiological tumor response rate, marked by complete pain relief (median onset within three doses) and rapid cortical re-mineralization and intralesional ossification (median onset within six doses) [8,16,24]. In the neoadjuvant setting, pre-operative denosumab successfully downstaged aggressive, locally destructive lesions in 7 out of 12 patients in the largest prospective cohort [24], converting radical segmental resections into conservative enucleation or curettage with complete giant cell depletion and woven bone formation on post-surgical histopathology. However, denosumab represents a temporary suppressive bridge rather than a curative monotherapy. Because it is purely osteoclast-suppressive, denosumab has zero effect on the proliferative, mutated mononuclear stromal cell compartment, which survives and resumes pathogenetic RANKL secretion upon drug clearance. Consequently, treatment discontinuation was followed by exceptionally high recurrence rates of up to 100% in pediatric cohorts [25] and 37% in mature adults [8,16]. In growing children, stopping denosumab provoked severe, life-threatening rebound hypercalcemia accompanied by acute kidney injury [31,32], necessitating post-therapy bisphosphonate bridges or the implementation of de-escalated weaning schedules. The summary of clinical outcomes across the 10 non-overlapping cohorts/case reports is presented with a note in Table 4.

**Figure 3:**
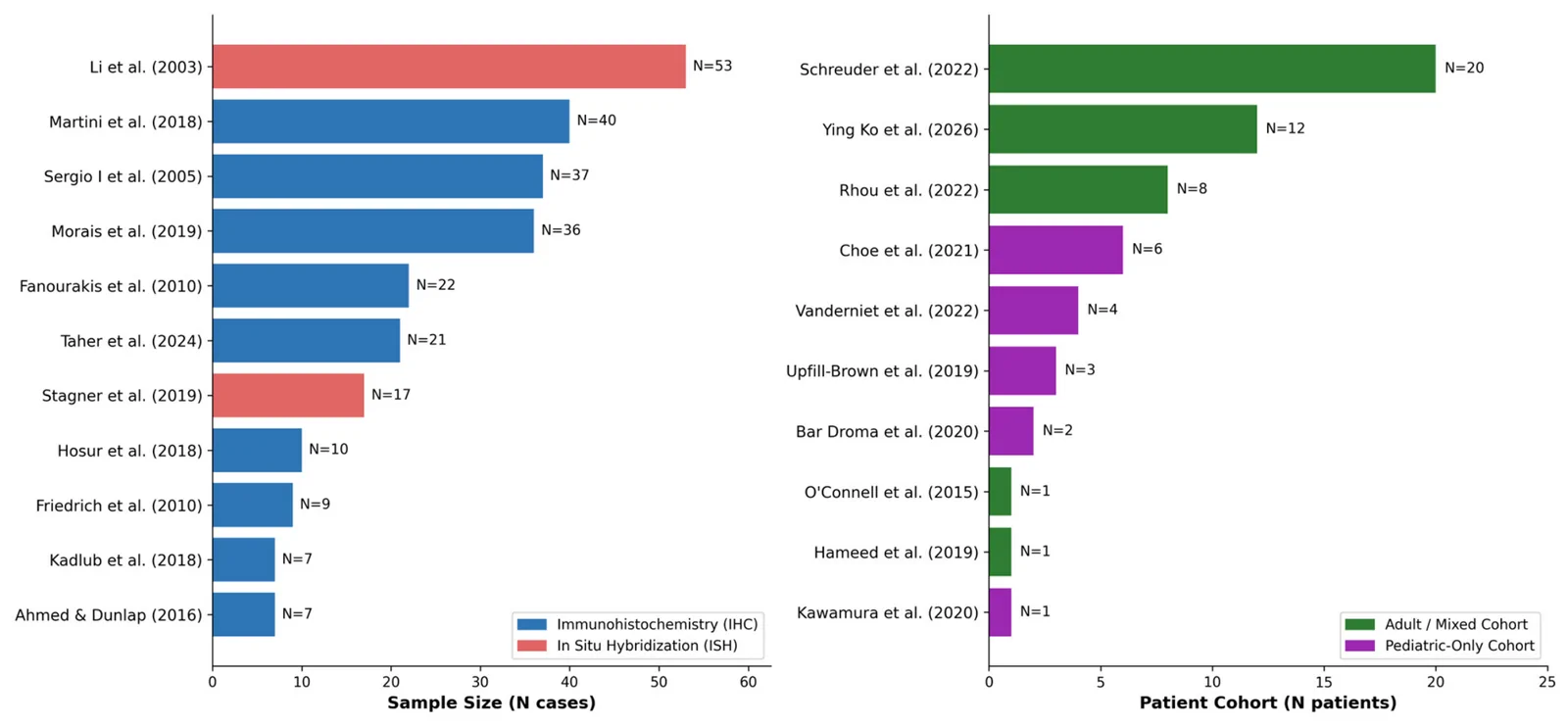
Visual evidence map and gap analysis of the included primary tissue-marker studies (**left**, N = 11 unique studies) and clinical denosumab therapy investigations (**right**, N = 10 unique studies), demonstrating data distribution and methodological transparency [3,4,5,6,7,8,16,21,24,25,26,27,28,29,30,31,32,33,34,35,36].

A consolidated evidence map and gap analysis across both the tissue-marker and clinical study cohorts is presented in Figure 3.

## 4. Discussion

The systematic integration of 11 primary tissue-marker studies establishes that gnathic giant cell-rich lesions represent a single pathologic spectrum driven by a common molecular denominator: the RANKL-mediated activation of osteoclastogenesis [4]. While clinical classification systems historically stratified CGCG, PGCG, cherubism, and ABC into distinct clinical boxes, tissue profiling demonstrates that they are genetically and pathologically unified [4]. In all these lesions, mature, multinucleated giant cells express classic osteoclastic cell-surface receptors (RANK) and proteolytic enzymes (TRAP, V-ATPase, Cathepsin K, MMP-9), resorbing jawbone tissue under the paracrine influence of proliferative, spindle-shaped mononuclear stromal cells that express high levels of the ligand RANKL [4,5].

A mechanistic hypothesis that merits discussion, although it is derived from a single external study rather than from the studies synthesized here, concerns the Secreted Osteoclastogenic Factor of Activated T Cells (SOFAT) axis [37]. SOFAT has been described as an activated human T cell-secreted cytokine reported to induce functional osteoclast formation and bone resorptive activity in a completely RANKL-independent and OP-resistant manner [37]. This evidence base is narrow: the SOFAT–osteoclastogenesis association rests on descriptive immunohistochemistry from a single small case series, without functional, genomic, or in vivo mechanistic validation; no published data currently link SOFAT expression to denosumab response in any giant cell lesion. Given this limited scientific evidence, the pathway should be regarded as a speculative avenue for future investigation rather than an established mechanism of treatment failure. Unlike canonical osteoclastogenesis, which demands cell-to-cell contact, SOFAT acts as a soluble cytokine that directly stimulates osteoblasts to secrete proinflammatory cytokines, primarily interleukin-6 (IL-6), which subsequently signal osteoclast precursor fusion [37]. In the comparative context of gnathic lesions, Cândido-Soares et al. [37] demonstrated that SOFAT is strongly immunopositive in the cytoplasm of mature giant cells within intraosseous lytic jaw lesions (CGCG, cherubism) but is completely absent in extraosseous reactive lesions (such as PGCG and foreign body reactions) [37]. This spatial restriction raises the possibility that the local immune microenvironment of intraosseous lesions may contain B- and T-lineage cell cascades that bypass the traditional RANK/RANKL/OPG pathway to perpetuate bone lysis [37]. Because SOFAT-mediated osteoclastogenesis was reported not to be inhibited by OPG in that experimental setting, it has been postulated that such a pathway would be insensitive to denosumab blockade [37]. If this were confirmed, a bypass axis of this kind could offer one possible partial explanation for why anti-RANKL therapy depletes early MGCs yet frequently fails to achieve permanent, non-surgical cures, culminating in the high metabolic recurrence rates observed upon drug discontinuation [8,37]. It must be emphasized, however, that this entire line of reasoning rests on one external immunohistochemical study [37], which did not examine denosumab-treated tissue and did not test any association between SOFAT expression and treatment response. No causal relationship between SOFAT activity and denosumab failure in gnathic giant cell lesions has been demonstrated; the proposal is advanced here strictly as a hypothesis. In-dependent validation in denosumab-treated gnathic cohorts is required before any clinical inference is drawn from this pathway.

This systematic review and meta-analysis bridges translational bone biology and organ-preserving clinical medicine by defining the critical therapeutic role of the RANK/RANKL/OPG pathway in jaw giant cell lesions. By establishing that proliferating, spindle-shaped mononuclear stromal cells drive osteolytic bone resorption through the secretion of RANKL to stimulate osteoclast precursors, this synthesis highlights denosumab (an anti-RANKL monoclonal antibody) as a key pharmacologic alternative to potentially mutilating jaw surgery [8,24]. While neoadjuvant denosumab can downstage aggressive lesions to enable conservative treatment, the review addresses critical post-treatment safety considerations by highlighting the high risk of recurrence upon therapy cessation, alongside the necessity of closely monitoring serum calcium levels in prepubertal patients to manage growth and post-treatment electrolyte imbalances [25,32]. To guide safe clinical practice and help to prevent post-cessation mineral surges—especially rebound hypercalcemia in growing pediatric skeletons—we propose the following as an expert-opinion protocol synthesized from the included studies in the literature, pending prospective validation, rather than as a direct statistical finding of this review: (1) a de-intensified monthly dosing protocol (120 mg without aggressive loading doses) to reduce early hypocalcemia risk [16,24]; (2) a stepwise weaning schedule that progressively spaces doses from 1 to 4 months in 1-month increments before complete cessation [32]; and (3) structured post-treatment multidisciplinary surveillance combining parathyroid hormone and serum calcium tracking with CBCT imaging at 3-month intervals for a minimum of 36 months [16,24]. These specific regimens have not been prospectively compared against alternatives in the included literature and should be validated in dedicated future trials before adoption as standard practice. To state this explicitly: the weaning and surveillance schedule proposed above is an expert-based opinion formed by the authors from the reports included in this review. It is not direct evidence generated by this review, no comparative data support one weaning schedule over another and prospective clinical validation is still needed before it is adopted in routine practice.

Several limitations within the existing literature must be acknowledged. First, the primary tissue expression studies are retrospective and limited by single-center archives; demographic matching is rarely performed (with the exception of Morais et al. [26]). Second, there is a substantial risk of false-negative diagnostic staining due to non-standardized decalcification pre-processing of bone biopsies, as hydrochloric acid can degrade both proteins and mRNA structures [27]. Third, the clinical therapy literature is comprised entirely of small cohorts and isolated case reports, lacking randomized clinical control groups [1]. Fourth, the quantitative meta-analysis pooled only two studies (k = 2), precluding formal heterogeneity assessment (I^2^, Cochran’s Q); the fixed-effect model was adopted for this reason rather than because homogeneity was demonstrated; both pooled estimates carry wide confidence intervals and should be interpreted as hypothesis-generating rather than definitive. Fifth, one clinical cohort (Bar Droma et al. [33]) included two related individuals (siblings), introducing non-independence into the pooled patient count. Sixth, Kawamura et al. [36] could not be formally risk-of-bias scored due to insufficient reported methodological detail and is, therefore, reported narratively only, which limits the completeness of the risk-of-bias tabulation in Table 2. Seventh, the overall base in the literature addressing this pathway in the jaws remains small in absolute terms (11 tissue-marker studies and 10 clinical investigations); the tissue-marker synthesis necessarily pools four biologically distinct entities—CGCG, PGCG, cherubism, and ABC—that likely differ in underlying pathogenetic mechanisms (reactive, monogenic, and clonal neoplastic, respectively) and natural biological behavior. Pooling across these entities for pathway-level synthesis may, therefore, mask entity-specific differences that a larger, disease-stratified evidence base could resolve. Eighth, immunolabeling across the included tissue studies was heterogeneous, with different primary antibody clones, staining protocols, and semi-quantitative scoring systems (e.g., SID scoring) applied inconsistently between centers; this methodological heterogeneity limits direct cross-study comparison of staining results and could not be adjusted for in the narrative synthesis. Finally, publication bias could not be formally assessed (e.g., via funnel plot), given the small number of eligible studies in both the tissue-marker and clinical syntheses.

**Recommendations for Future Research**

Building on the limitations identified above, we propose the following priorities for future research. First, prospective, multi-institutional cohort studies with standardized RANK/RANKL/OPG staining and reporting protocols (e.g., harmonized staining-intensity distribution scoring or digital image quantification) are needed to replace the current retrospective, single-center evidence base and to enable demographic matching between aggressive and non-aggressive phenotypes, as this was achieved by only one included study to date. Second, tissue-processing protocols should be validated and standardized to minimize decalcification-related antigen and mRNA degradation, reducing the risk of false-negative staining in future studies. Third, adequately powered comparative studies are needed to formally test RANK expression differences between aggressive and non-aggressive CGCG; the present meta-analysis pooled only two studies and could not assess heterogeneity. Therefore, its estimates should be treated as hypothesis-generating rather than confirmatory. Fourth, prospective trials are warranted to formally evaluate de-escalated and stepwise denosumab-weaning regimens, as the dosing and monitoring protocol proposed in this review is a synthesis of expert opinion rather than a prospectively validated finding. Fifth, dedicated longitudinal surveillance cohorts are needed to define the natural history and optimal monitoring interval for post-cessation recurrence and pediatric rebound hypercalcemia, including systematic tracking of parathyroid hormone and bone turnover markers, which were inconsistently reported across the included clinical studies. Finally, independent replication is needed to confirm the role of the RANKL-independent, OPG-resistant SOFAT pathway in gnathic giant cell lesions specifically, since this hypothesis currently rests on a single tissue study; confirmatory work could clarify whether SOFAT activity explains treatment failure and identify candidate lesions less likely to respond to anti-RANKL therapy alone.

## 5. Conclusions

This systematic review and meta-analysis indicates that the RANK/RANKL/OPG signaling pathway is a primary pathogenetic driver of osteolytic destruction in gnathic giant cell lesions, with mononuclear stromal cell-derived RANKL driving the osteoclastogenesis of RANK-expressing giant cells. The quantitative meta-analysis, however, pooled only two studies and showed no statistically significant difference in RANK expression between aggressive and non-aggressive CGCG; the borderline SID-score result (*p* = 0.0506, with a 95% CI crossing zero) is a non-significant trend rather than a confirmed difference, and should be read as hypothesis-generating. While denosumab represents a highly effective neoadjuvant option to downstage surgical morbidity, the high post-cessation recurrence and severe post-treatment rebound hypercalcemia in children indicate that the therapy does not eliminate the latent, proliferative stromal precursor population. A SOFAT-mediated bypass axis is tentatively hypothesized as an additional, RANKL-independent and OPG-resistant bypass pathway that may help to explain the high metabolic relapse rates observed upon drug discontinuation, although this hypothesis rests on a single external tissue study [37], has not been tested in denosumab-treated gnathic lesions, and warrants independent validation before it is used to inform clinical decision-making. Based on the safety signals identified in this review, we suggest, as an expert-opinion protocol pending prospective validation, not as a direct finding of this synthesis, that clinicians consider standardized tissue staining protocols, close long-term follow-up, and de-escalated weaning schedules to help to maintain mineral homeostasis and optimize jaw preservation.

**Clinical Relevance:** This review indicates that jaw giant cell lesions share a common RANKL-driven pathogenetic mechanism, supporting the rationale for neoadjuvant denosumab to help to avoid disfiguring resection. However, clinicians should anticipate a substantial risk of recurrence after treatment cessation and should implement structured post-treatment metabolic monitoring, particularly in pediatric patients, given the risk of rebound hypercalcemia. Clinicians should also note that denosumab is not licensed for gnathic giant cell lesions in most territories and is, therefore, used off-label for this indication; thus, its use should follow local off-label prescribing governance and informed consent requirements. The de-escalated dosing and weaning schedule proposed in this review is, likewise, expert-based opinion pending prospective validation, not a directly evidenced finding.

## Figures and Tables

**Table 1:** Summary of key findings of included tissue-marker studies.

Study ID and Design	Population/N	Method	Markers	Primary Pathogenetic Findings
Li et al. (2003) Comparative [5]	CGCG (34), PGCG (6), Cherubism (7), ABC (6)	ISH and IHC	RANK, RANKL, OPG	RANKL mRNA localized to spindle MSCs; OPG mRNA in MGCs and MSCs; RANK mRNA in MGCs. MGCs are phenotypic osteoclasts.
Sergio et al. (2005) Comparative [3]	CGCG (37) [27 Agg, 10 Non-Agg]	IHC	RANK, GRα, CTR	MGCs and MSCs moderately express RANK. No significant difference in RANK between aggressive and non-aggressive subtypes. SID score trended higher in non-aggressive cases. Significant correlation between RANK staining and clinical aggressiveness was not found.
Fanourakis et al. (2010) Descriptive [7]	PGCG (22)	IHC	RANKL, OPG	OPG and RANKL expression detected in spindle stromal cells. Staining pattern was consistent with osteoblastic lineage, actively mediating MGC fusion and recruitment. Active giant cells exhibit intense reactivity for both proteins.
Morais et al. (2019) Comparative [26]	CGCG (10), PGCG (13), peri-implant PGCG (13)	IHC	CD68, OPG, RANK	RANK and OPG scores similar across cohorts. CD68 and Bcl-2 higher in conventional jaw lesions.
Stagner et al. (2019) Comparative [27]	CGCL (17) [10 GCT-like, 7 non-GCT]	RNA-ISH	RANKL mRNA	Eighty-eight percent of tumors strongly express RANKL in mononuclear stromal cells, regardless of aggressive behavior or GCT-like histopathology.
Taher et al. (2024) Comparative [21]	CGCG (21) [9 Agg, 12 Non-Agg]	IHC	RANK, Osteocalcin	Non-aggressive CGCGs had significantly higher RANK SID scores (*p* = 0.021). Aggressive CGCGs had significantly higher OC (*p* = 0.023).
Kadlub et al. (2018) Descriptive [28]	Cherubism (N = 7)	IHC	RANKL, OPG, RANK	First comprehensive analysis in cherubism tissue, proving that SH3BP2 driver mutations induce massive stromal RANKL expression and NFATc1 nuclear translocation.
Ahmed and Dunlap (2016) Descriptive [6]	GCG of jaws (7)	IHC	RANKL, c-fos, p53	All tissues showed strong, diffuse nuclear c-fos and cytoplasmic RANKL in MSCs and MGCs. p53 was negative.
Martini et al. (2018) Comparative [4]	CGCG (20), PGCG (20)	IHC	RANKL, OPG	Number of MGC nuclei significantly higher in CGCG (*p* = 0.010). RANKL expression significantly higher in CGCG than PGCG (*p* = 0.042).
Hosur et al. (2018) Descriptive [29]	CGCG (10)	IHC	RANK, RANKL, p63	Confirms diffuse, strong RANK/RANKL staining in CGCG, while p63 negativity successfully distinguishes CGCG from true giant cell tumor (GCT) of bone.
Friedrich et al. (2010) Descriptive [30]	CGCG (4), PGCG (5)	IHC	RANK, CD68, CD51	Immunolabeled CD51 and CD68 alongside RANK, verifying that multinucleated giant cells represent functional, active bone-resorbing osteoclasts.

**Table 2:** Methodological quality and risk-of-bias assessment (consolidated N = 21 studies).

Study ID	Study Design	Assessment Tool	Score/Stars Met	Primary Methodological Limitations and Gaps
**PART A: TISSUE STUDIES**				
Li et al. (2003) [5]	Comparative	NOS Scale	7/9 stars **(Low Risk)**	Did not match cohorts for age/gender; decalcification protocol details omitted.
Sergio et al. (2005) [3]	Comparative	NOS Scale	6/9 stars **(Low Risk)**	Unmatched cohorts; lack of double-blind pathological scoring of IHC.
Fanourakis et al. (2010) [7]	Case Series	JBI Checklist	8/10 (80%) **(Low Risk)**	Small mucosal sample (N = 22); retrospective clinical selection bias; no blood OPG controls.
Morais et al. (2019) [26]	Comparative	NOS Scale	9/9 stars **(Low Risk)**	Excellent matching for age and site but limited by retrospectivity and fixative variations.
Stagner et al. (2019) [27]	Case Series	JBI Checklist	7/10 (70%) **(Mod Risk)**	Retrospective selection bias (82% aggressive cases); decalcification has high false-negative risk.
Taher et al. (2024) [21]	Comparative	NOS Scale	6/9 stars **(Low Risk)**	Small sample volume (N = 21); unmatched cohorts; lack of longitudinal bone marker correlates.
Kadlub et al. (2018) [28]	Descriptive	JBI Checklist	8/10 **(Low Risk)**	Lacks details on consecutive clinical inclusion; retrospective tissue retrieval.
Ahmed and Dunlap (2016) [6]	Case Series	JBI Checklist	6/10 (60%) **(Mod Risk)**	Extremely small sample size (N = 7); completely descriptive study with no statistical controls.
Martini et al. (2018) [4]	Comparative	NOS Scale	8/9 stars **(Low Risk)**	Retrospective design; lacked clinical follow-up data to correlate staining with true recurrence.
Friedrich et al. (2010) [30]	Descriptive	JBI Checklist	7/10 (70%) (Mod Risk)	Anatomically heterogeneous comparison; small gnathic subset limits power.
Hosur et al. (2018) [29]	Descriptive	JBI Checklist	7/10 (70%) (Mod Risk)	Small retrospective cohort (N = 10); lacked longitudinal clinical recurrence data.
**PART B: CLINICAL STUDIES**				
Ying Ko et al. (2026) [24]	Prospective Cohort	JBI Case Series	9/10 (90%) **(Low Risk)**	Single center; lacks random comparison; one patient was a pediatric crossover.
Schreuder et al. (2022) [8]	Retrospective Cohort	JBI Case Series	9/10 (90%) **(Low Risk)**	Retrospective multicenter; varying CT follow-up intervals; selection bias.
Rhou et al. (2022) [16]	Prospective Observation	JBI Case Series	9/10 (90%) **(Low Risk)**	Small cohort size (N = 8) limits statistical power; lacks a control group.
Vanderniet et al. (2022) [25]	Pediatric Series	JBI Case Series	8/10 (80%) **(Low Risk)**	Extremely small sample (N = 4); missing longitudinal follow-up for bone growth plates.
Choe et al. (2021) [31]	Pediatric Series	JBI Case Series	7/10 (70%) **(Mod Risk)**	Retrospective pediatric sample (N = 6); non-standardized post-treatment calcium monitoring.
Upfill-Brown et al. (2019) [32]	Pediatric Series	JBI Case Series	8/10 (80%) **(Low Risk)**	Heterogeneous pediatric cohort (ABC, CGCG, Cherubism); lack of baseline PTH and osteocalcin.
Bar Droma et al. (2020) [33]	Pediatric Series	JBI Case Series	7/10 (70%) **(Mod Risk)**	N = 2 pediatric sisters; retrospectively reviewed; sparse serum monitor records.
Hameed et al. (2019) [34]	Single Case Report	JBI Case Report	7/8 (87.5%) **(Low Risk)**	Descriptive N = 1 case; lacked post-cessation biochemical surveillance data.
O’Connell et al. (2015) [35]	Single Case Report	JBI Case Report	8/8 (100%) **(Low Risk)**	Potential confounding effect from prior long-term intralesional steroid injections.
Kawamura et al. (2020) [36]	Single Case Report	JBI Case Report	8/8 (100%) **(Low Risk)**	Single-case design; short 6-month treatment duration; no generalizability.

**Table 3:** Cellular and compartmentalized staining localization of RANK/RANKL/OPG (N = 11).

Study ID	RANK Localization	RANKL Localization	OPG Localization
Sergio et al. (2005) [3]	MGC cell membrane; peripheral stromal cell borders.	Not evaluated.	Not evaluated.
Taher et al. (2024) [21]	MGC membrane and cytoplasm; focal in stromal cells.	Not evaluated.	Not evaluated.
Martini et al. (2018) [4]	Not evaluated.	Proliferating MSC cytoplasm; MGC peripheral ring.	Mononuclear stromal cells; weakly positive in some MGC borders.
Morais et al. (2019) [26]	Not evaluated.	Strong cytoplasm of peri-implant stromal cells.	Mononuclear cell cytoplasm; highly variable in giant cells.
Li et al. (2003) [5]	MGC cytoplasmic transcripts; high in osteoclast precursors.	Strong mRNA in spindle-shaped mononuclear stromal cells.	Abundant transcripts within active osteoblastic and stromal bands.
Stagner et al. (2019) [27]	Not evaluated.	RNA scope shows transcripts restricted to stromal MSCs.	Not evaluated.
Fanourakis et al. (2010) [7]	Not evaluated.	Intense cytoplasmic staining of stromal cell populations.	Mononuclear stromal cells; completely negative in giant cell cores.
Ahmed and Dunlap (2016) [6]	Not evaluated.	Diffuse cytoplasmic staining of stromal cells.	Not evaluated.
Kadlub et al. (2018) [28]	Strong in MGC cytoplasm and osteoclast precursors.	Highly positive in mutated SH3BP2 stromal populations.	Weakly positive in background fibrous bands; absent in GCs.
Friedrich et al. (2010) [30]	MGC membranes; strongly positive in active bone zones.	Not evaluated.	Not evaluated.
Hosur et al. (2018) [29]	Strongly positive on active multinucleated giant cell borders.	Diffuse cytoplasm of mononuclear stromal cells.	Not evaluated.

**Table 4:** Summary of clinical outcomes and regimens of denosumab therapy (10 non-overlapping cohorts/case reports; N = 58 patients) Study type is stated for every entry: seven multi-patient cohorts (55 patients) and three single-case reports (3 patients).

Study ID	Population (N)	Dosing Regimen	Clinical/Surgical Outcome	Recurrence and Adverse Events
Ying Ko et al. (2026) [24]—multi-patient cohort	Adult/Adolescent (N = 12)	70 mg/m^2^ or 120 mg flat; monthly. Avoided loading doses.	100% radiographic response. Nine patients received conservative curettage instead of resection.	No recurrences in neoadjuvant group. Spacing intervals mitigated rebound hypercalcemia.
Schreuder et al. (2022) [8]—multi-patient cohort	Adult/Adolescent (N = 20)	120 mg flat; weekly loading, then monthly for 6–12 months.	A 100% volumetric and metabolic complete/partial response. Pre-op downstaging in maxilla/mandible.	High metabolic recurrence (37% to 100%) peak at 12 months post cessation. Low risk of jaw osteonecrosis.
Rhou et al. (2022) [16]—multi-patient cohort	Adult/Adolescent (N = 8)	120 mg flat; standard loading doses.	Excellent local ossification and metabolic remission (documented by PET-CT).	Metabolic recurrence caught early by PET-CT surveillance in three patients.
Vanderniet et al. (2022) [25]—multi-patient cohort	Pediatric (N = 4)	70 mg/m^2^; monthly, followed by 2 zoledronate doses.	Complete bone consolidation, allowing for conservative dental-preserving enucleation.	100% metabolic recurrence on PET-CT; severe rebound hypercalcemia requiring bisphosphonates.
Choe et al. (2021) [31]—multi-patient cohort	Pediatric (N = 6)	120 mg; standard dose adjusted for skeleton.	Significant mineral density increase in CT; complete surgical preservation of jaw contour.	Moderate post-cessation recurrence; two children developed moderate hypocalcemia.
Upfill-Brown et al. (2019) [32]—multi-patient cohort	Pediatric (N = 3)	70 mg/m^2^; weekly loading, then monthly.	Marked bone consolidation, resolving active mandibular border-perforation.	Severe hypercalcemia (vomiting, dehydration) peaking 4–5 months after drug cessation.
Bar Droma et al. (2020) [33]—multi-patient cohort	Pediatric (N = 2 sisters)	70 mg/m^2^; monthly in cherubism.	Dramatically resolved airway obstruction and stabilized tooth displacement.	Asymptomatic hypocalcemia during treatment; a progressive weaning interval controlled the hypercalcemia.
Hameed et al. (2019) [34]—single-case report	Adult/Adolescent (N = 1 case)	120 mg flat; standard protocol.	Complete jaw consolidation. Prevented disfiguring mandibular reconstruction.	No recurrence reported at short 12-month follow-up.
O’Connell et al. (2015) [35]—single-case report	Adult/Adolescent (N = 1 case)	120 mg; pre-operative neoadjuvant.	Downstaged a massive, aggressive intraosseous mass to conservative tooth-preserving curettage.	Stable bone consolidation; no post-treatment recurrence at 24 months.
Kawamura et al. (2020) [36]—single-case report	Pediatric (N = 1 case)	70 mg/m^2^; monthly for cherubism.	Complete lesion regression and rapid bone re-mineralization of the mandible.	Weaning scheduling successfully avoided mineral/electrolyte rebound crises.

## Data Availability

The extracted study-level data underlying Table 1, Table 2, Table 3 and Table 4 and the meta-analysis input data (means, standard deviations, and sample sizes for the two pooled studies underlying Figure 2) are included within this article. No original datasets, collection forms, or analytic code beyond standard meta-analysis formulas were generated; any further extraction materials are available from the corresponding author upon reasonable request.

## Supplementary Materials

The following supporting information can be downloaded at https://www.mdpi.com/article/10.3390/diseases14100360/s1, File S1: PRISMA_2020_checklist; File S2: Full Search Strategies.

## Abbreviations

CGCG	Central giant cell granuloma
PGCG	peripheral giant cell granuloma
ABCs	aneurysmal bone cysts
RANK	Receptor activator of nuclear factor-κB
RANKL	Receptor activator of nuclear factor-κB ligand
OPG	osteoprotegerin
NOS	Newcastle-Ottawa Scale
JBI	Joanna Briggs Institute
SOFAT	Secreted Osteoclastogenic Factor of Activated T Cell
MGCs	Multinucleated giant cells
MSCs	Mononuclear stromal cells
RoB	Risk of bias
IHC	Immunohistochemistry
ISH	In situ hybridization
SID	Staining intensity distribution score
