# Supplementary material for: RANK/RANKL/OPG Axis Expression and Response to Anti-RANKL Therapy in Gnathic Giant Cell Lesions: A Systematic Review and Meta-Analysis
Source: Diseases. 2026 Oct 1;14(10):360. doi: 10.3390/diseases14100360 (PMC13703804; doi:10.3390/diseases14100360)
Supplement: Supplementary file 1 [file diseases-14-00360-s001.zip › supplement material file S1.pdf]

PRISMA 2020 Checklist

Section and Topic	Item #	Checklist item	Location where item is reported
**TITLE**			
Title	1	Identify the report as a systematic review.	Title
**ABSTRACT**			
Abstract	2	See the PRISMA 2020 for Abstracts checklist.	Abstract (structured: Background/Objectives, Methods, Results, Conclusions)
**INTRODUCTION**			
Rationale	3	Describe the rationale for the review in the context of existing knowledge.	Introduction, para. 1–3
Objectives	4	Provide an explicit statement of the objective(s) or question(s) the review addresses.	Introduction, final paragraphs
**METHODS**			
Eligibility criteria	5	Specify the inclusion and exclusion criteria for the review and how studies were grouped for the syntheses.	2.3 Eligibility Criteria
Information sources	6	Specify all databases, registers, websites, organisations, reference lists and other sources searched or consulted to identify studies. Specify the date when each source was last searched or consulted.	2.1 (PubMed/MEDLINE, Scopus, Web of Science; inception–Aug 2025, updated to Jul 2026)
Search strategy	7	Present the full search strategies for all databases, registers and websites, including any filters and limits used.	2.1); full Boolean strings for PubMed/MEDLINE, Scopus, and Web of Science, including denosumab sub-question terms, provided in Supplementary Material S1
Selection process	8	Specify the methods used to decide whether a study met the inclusion criteria of the review, including how many reviewers screened each record and each report retrieved, whether they worked independently, and if applicable, details of automation tools used in the process.	2.3 (duplicate independent screening and disagreement-resolution process stated; inter-rater agreement statistics explicitly stated as not calculated)
Data collection process	9	Specify the methods used to collect data from reports, including how many reviewers collected data from each report, whether they worked independently, any processes for obtaining or confirming data from study investigators, and if applicable, details of automation tools used in the process.	2.1 (explicit statement: duplicate, independent data extraction using a standardized pre-piloted form; disagreements resolved by discussion/third-author adjudication)
Data items	10a	List and define all outcomes for which data were sought. Specify whether all results that were compatible with each outcome domain in each study were sought (e.g. for all measures, time points, analyses), and if not, the methods used to decide which results to collect.	2.2 PECO/PICO framework, Outcome row
	10b	List and define all other variables for which data were sought (e.g. participant and intervention characteristics, funding sources). Describe any assumptions made about any missing or unclear information.	2.2 PECO/PICO framework, Population/Intervention rows
Study risk of bias assessment	11	Specify the methods used to assess risk of bias in the included studies, including details of the tool(s) used, how many reviewers assessed each study and whether they worked independently, and if applicable, details of automation tools used in the process.	2.4 (NOS domains used and rationale for “adaptation” now stated; RoB scoring disagreement-resolution process stated; inter-rater agreement statistics explicitly stated as not calculated
Effect measures	12	Specify for each outcome the effect measure(s) (e.g. risk ratio, mean difference) used in the synthesis or presentation of results.	2.5 (Hedges' g, standardized mean difference)
Synthesis	13a	Describe the processes used to decide which studies were eligible for	2.5 (reframed as a pre-specified general rule: only studies with directly comparable

Section and Topic	Item #	Checklist item	Location where item is reported
methods		each synthesis (e.g. tabulating the study intervention characteristics and comparing against the planned groups for each synthesis (item #5)).	staining/scoring protocols are pooled quantitatively)
	13b	Describe any methods required to prepare the data for presentation or synthesis, such as handling of missing summary statistics, or data conversions.	2.5 (states means/SDs were extracted directly as reported; no imputation or conversion was needed)
	13c	Describe any methods used to tabulate or visually display results of individual studies and syntheses.	Tables 1–4; Figures 2–3
	13d	Describe any methods used to synthesize results and provide a rationale for the choice(s). If meta-analysis was performed, describe the model(s), method(s) to identify the presence and extent of statistical heterogeneity, and software package(s) used.	2.5 (fixed-effect inverse-variance model in RevMan v5.4; heterogeneity not estimable at k=2, stated explicitly)
	13e	Describe any methods used to explore possible causes of heterogeneity among study results (e.g. subgroup analysis, meta-regression).	2.5 (heterogeneity exploration not applicable at k=2, stated explicitly)
	13f	Describe any sensitivity analyses conducted to assess robustness of the synthesized results.	2.5 (sensitivity analyses not applicable/not conducted, stated explicitly)
Reporting bias assessment	14	Describe any methods used to assess risk of bias due to missing results in a synthesis (arising from reporting biases).	New Methods paragraph after §2.5 (states formal assessment of missing-results bias was not performed and why, prospectively)
Certainty assessment	15	Describe any methods used to assess certainty (or confidence) in the body of evidence for an outcome.	New Methods paragraph after §2.5 (states no formal certainty/GRADE assessment was performed and why)
**RESULTS**			
Study selection	16a	Describe the results of the search and selection process, from the number of records identified in the search to the number of studies included in the review, ideally using a flow diagram.	Figure 1 (PRISMA flow diagram) + §3.1 narrative
	16b	Cite studies that might appear to meet the inclusion criteria, but which were excluded, and explain why they were excluded.	3.1 gives exclusion category counts (reviews, animal/in vitro, duplicate thesis, small case reports, unpublished dissertations); individual excluded-study citations not itemized
Study characteristics	17	Cite each included study and present its characteristics.	Table 1 (tissue studies); Table 4 (clinical studies)
Risk of bias in studies	18	Present assessments of risk of bias for each included study.	3.2 and Table 2 ; all included.
Results of individual studies	19	For all outcomes, present, for each study: (a) summary statistics for each group (where appropriate) and (b) an effect estimate and its precision (e.g. confidence/credible interval), ideally using structured tables or plots.	Tables 1 and 4 give findings; full per-study effect estimates with CIs given only for the 2 meta-analyzed studies
Results of syntheses	20a	For each synthesis, briefly summarise the characteristics and risk of bias among contributing studies.	3.4–3.5 narrative
	20b	Present results of all statistical syntheses conducted. If meta-analysis was done, present for each the summary estimate and its precision	Figure 2 (forest plots with pooled Hedges' g, 95% CI, heterogeneity note)

PRISMA 2020 Checklist

Section and Topic	Item #	Checklist item	Location where item is reported
		(e.g. confidence/credible interval) and measures of statistical heterogeneity. If comparing groups, describe the direction of the effect.	
	20c	Present results of all investigations of possible causes of heterogeneity among study results.	3.4 Results (states heterogeneity exploration not applicable at k=2)
	20d	Present results of all sensitivity analyses conducted to assess the robustness of the synthesized results.	3.4 Results (states sensitivity analyses not applicable/not conducted)
Reporting biases	21	Present assessments of risk of bias due to missing results (arising from reporting biases) for each synthesis assessed.	3.4 Results (states risk of bias due to missing results not assessed for this synthesis)
Certainty of evidence	22	Present assessments of certainty (or confidence) in the body of evidence for each outcome assessed.	3.4 Results (states no certainty/GRADE assessment performed for this synthesis)
**DISCUSSION**			
Discussion	23a	Provide a general interpretation of the results in the context of other evidence.	4 Discussion, opening paragraphs
	23b	Discuss any limitations of the evidence included in the review.	4 Limitations paragraph
	23c	Discuss any limitations of the review processes used.	4 Limitations paragraph (k=2 meta-analysis, small evidence base, exclusions)
	23d	Discuss implications of the results for practice, policy, and future research.	Clinical Relevance statement; Recommendations for Future Research section
**OTHER INFORMATION**			
Registration and protocol	24a	Provide registration information for the review, including register name and registration number, or state that the review was not registered.	Abstract; 2.1 (PROSPERO ID; format pending author verification)
	24b	Indicate where the review protocol can be accessed, or state that a protocol was not prepared.	2.1 (PROSPERO record stated as publicly accessible at crd.york.ac.uk/prospero/)
	24c	Describe and explain any amendments to information provided at registration or in the protocol.	2.1 (“No amendments were made to the registered protocol or eligibility criteria after registration” — author to confirm this is accurate)
Support	25	Describe sources of financial or non-financial support for the review, and the role of the funders or sponsors in the review.	7 Funding ("funded entirely by the authors")
Competing interests	26	Declare any competing interests of review authors.	11 Declaration of competing interest
Availability of data, code and other materials	27	Report which of the following are publicly available and where they can be found: template data collection forms; data extracted from included studies; data used for all analyses; analytic code; any other materials used in the review.	6 Data availability statement (now itemizes what is included in the article vs. available on request)

From: Page MJ, McKenzie JE, Bossuyt PM, Boutron I, Hoffmann TC, Mulrow CD, et al. The PRISMA 2020 statement: an updated guideline for reporting systematic reviews. BMJ 2021;372:n71. doi: 10.1136/bmj.n71.
This work is licensed under CC BY 4.0. To view a copy of this license, visit https://creativecommons.org/licenses/by/4.0/
