# Supplementary material for: RANK/RANKL/OPG Axis Expression and Response to Anti-RANKL Therapy in Gnathic Giant Cell Lesions: A Systematic Review and Meta-Analysis
Source: Diseases. 2026 Oct 1;14(10):360. doi: 10.3390/diseases14100360 (PMC13703804; doi:10.3390/diseases14100360)
Supplement: Supplementary file 1 [file diseases-14-00360-s001.zip › supplement material file S2.pdf]

Supplementary Material File S2: Full Search Strategies

PubMed/MEDLINE (searched from inception to July 2026)

("RANK" OR "receptor activator of nuclear factor kappa B" OR "RANKL" OR "receptor activator of nuclear factor kappa B ligand" OR "osteoprotegerin" OR "OPG") AND ("central giant cell granuloma" OR "giant cell granuloma" OR "giant cell lesion of the jaw" OR "peripheral giant cell granuloma" OR "cherubism" OR "aneurysmal bone cyst") AND ("jaw" OR "mandible" OR "maxilla" OR "gnathic")

Filters: none applied beyond date range; no language restriction (non-English records screened via translation).

Scopus

TITLE-ABS-KEY ((rank OR rankl OR "receptor activator of nuclear factor kappa B ligand" OR osteoprotegerin OR opg) AND ("central giant cell granuloma" OR "giant cell granuloma" OR "peripheral giant cell granuloma" OR cherubism OR "aneurysmal bone cyst" OR "giant cell lesion") AND (jaw OR mandible OR maxilla OR gnathic))

Web of Science (Core Collection)

TS=((RANK OR RANKL OR osteoprotegerin OR OPG) AND ("central giant cell granuloma" OR "giant cell granu-loma" OR "peripheral giant cell granuloma" OR cherubism OR "aneurysmal bone cyst") AND (jaw OR mandible OR maxilla OR gnathic))

Denosumab / clinical therapy sub-question terms

Combined with the marker/lesion terms above using AND: (denosumab OR "anti-RANKL therapy" OR "RANKL inhibitor" OR "RANKL inhibition")
